# Healthy Aging 2.0: The Potential of New Media and Technology

**Published:** 2012-03-08

**Authors:** Amanda K. Hall, Michael Stellefson, Jay M. Bernhardt

**Affiliations:** University of Florida, Department of Health Education and Behavior, Gainesville, Florida; University of Florida, Department of Health Education and Behavior, Gainesville, Florida; University of Florida, Department of Health Education and Behavior

## Introduction

The emergence of e-patients (consumers who use the Internet and electronic communication tools to research and communicate about medical conditions) has spawned the era of "Healthy Aging 2.0" to support chronic disease management. Approximately 125 million Americans are living with 1 or more chronic diseases, and this number is expected to grow to 157 million by 2020 (1). Approximately 84% of adults who are aged 65 or older have 1 or more chronic conditions (1). Healthy Aging 2.0 proposes that 21st century information and communications technology offers public health practitioners the unique opportunity to empower, engage, and educate these older adults in chronic disease management.

## Telemedicine

The power of telemedicine technologies to harness the capability of existing health care systems can help sustain the overall public health infrastructure. Telemedicine is "the delivery of health care services, where distance is a critical factor, by all health care professionals using information and communication technologies for the exchange of valid information for diagnosis, treatment, and prevention of disease and injuries" (2). Various innovations in remote medicine have demonstrated viability for monitoring chronic illness in cost-effective ways. Specifically, the European Commission noted that telemedicine could meet special needs of older adults, allowing them to live longer in their own homes, with more independence, and reduce the costs for inpatient care (3). For example, telemedicine devices that monitor chronic disease patients' vital signs (eg, blood pressure, weight, pulse oximetry) can cut health care costs and prevent hospitalizations (4,5).

Telemedicine platforms support interactive information sharing between patients and providers. Technologies such as smartphones and voice-over Internet protocol software applications (eg, Skype) can bridge geographic gaps between hard-to-reach chronic disease patients and their health care professionals. The Gary and Mary West Wireless Health Institute in San Diego, for instance, is developing wireless tools such as smart pills that can be monitored as patients swallow them. The SmartPill Corporation developed the first ambulatory diagnostic tool for gastrointestinal disorders, which uses sensors to measure pH, pressure, and temperature within the gastrointestinal tract (Figure). Another innovation, the iShoe*,* monitors how people walk and allows health practitioners to determine risk for falls. These types of interoperable, user-centered design features provide real-time information exchange between patients and providers, resulting in enhanced health communication where distance and access is a challenge.

**Figure.**
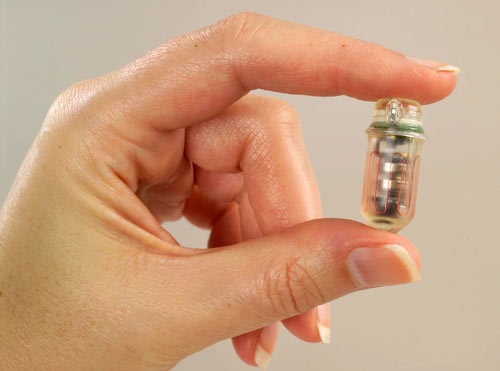
SmartPill. The SmartPill Wireless Motility Capsule is an ingestible device that uses sensors to measure pH, pressure, and temperature from within the gastrointestinal tract and sends information wirelessly to a remote server for health care professionals to assess. Reprinted with permission of the SmartPill Corporation. Photo by Donna Coveney.

## Web 2.0 and Internet Access

Chronic disease patients are taking a more active approach to managing their health by using interactive web 2.0 technologies. Web 2.0 technologies are highly participatory and have great potential for keeping older patients connected and informed. Chronic disease patients are using the Internet to seek information to improve their condition and make health decisions (6). Approximately 75% of all e-patients with a chronic condition reported that a previous health information search contributed to a decision about how to treat an ailment, and 69% said that the information prompted new questions to ask doctors (6). Use of the Internet continues to rise among older adults (age ≥65); in fact, significant increases have been noted in the last decade (7). According to a 2009 US Census report, however, only 42% of older adults actively access the Internet, and just 53% live in households with Internet access (8). Given the less than optimal frequency of Internet use, Hughes et al (9) advocate for "the use of a specific set of [web 2.0] tools by actors in health care including doctors, patients, and scientists, using principles of open source and generation of content by users, and the power of networks in order to personalize health care, collaborate, and promote health education."

A rise in Internet use by e-patients has paralleled a rise in social networking among older adults (10). A 2010 survey by the Pew Internet and American Life Project noted that 26% of older adults who used the Internet reported using social media (eg, Facebook, YouTube, Twitter), up 13% from 2009. This growth is expected to continue (10). The use of social media encourages information sharing and increased connectivity among older adults, which offers chronic disease patients new and valuable channels for social support and patient engagement (10). The incorporation of social networking applications in public health has increased the potential for more remote, shared decision making and more effective, tailored health information dissemination. For example, HealthTap offers secure mobile access to a social network of more than 5,000 physicians where patients can ask questions about their health concerns and receive customized answers from health care experts. Another social networking site, PatientsLikeMe, connects patients with other patients to share treatment information and experiences for common illnesses and disorders.

Researchers are beginning to examine which psychosocial and structural barriers prevent older adults from using the Internet. A study from the Center for Research and Education on Aging and Technology Enhancement found that some older adults have anxiety and low self-efficacy with computers and the Internet (11). Furthermore, Morrell et al found that lack of access to technology and lack of knowledge were primary reasons older adults did not use the Internet (12). In light of these research findings, training programs should be developed to reduce the anxiety older adults may feel about retrieving online health information and also increase demand for online health information by reducing barriers to access.

## Mobile Devices

"mHealth" is the use of mobile devices and technology for health by consumers and health care professionals. The concept of "mHealthy Aging" proposes that wireless tools can be used to promote healthy aging in the home. mHealthy Aging tools can help prevent isolation and neglect and also improve overall patient-provider communication. For instance, Trumpia is a cellular telephone reminder tool to support older adults in taking their medications as prescribed. Sensei Wellness is an mHealth application that offers personalized digital support, tracking, and feedback of users' health-related activities to encourage personal health surveillance. The potential benefits of mHealth tools are numerous, but a challenge will be finding ways to incorporate evidence-based, scalable, and usable mHealthy Aging tools into public health research and practice.

## Conclusion

New media and technology allow older adult e-patients, especially those with chronic conditions, more opportunity to access health information, receive online support, and engage health care professionals for disease management support in their homes. The evolution of e-patients and mHealth tools and the use of social networking and telemedicine interventions have led to opportunities to achieve better chronic disease outcomes. These interactive, technologically mediated interventions can empower, engage, and educate older adults. The systems integration opportunities presented through social networking and mHealth technologies will greatly contribute to a new era of Healthy Aging 2.0; moreover, the effective use of participatory technologies can revolutionize chronic disease care among older adult populations.
